# Minimum lesion detectability as a measure of PET system performance

**DOI:** 10.1186/s40658-017-0179-2

**Published:** 2017-03-04

**Authors:** Stephen Adler, Jurgen Seidel, Peter Choyke, Michael V. Knopp, Katherine Binzel, Jun Zhang, Craig Barker, Shielah Conant, Roberto Maass-Moreno

**Affiliations:** 1 Clinical Research Directorate/Clinical Monitoring Research Program, Leidos Biomedical Research, Inc., Frederick National Laboratory for Cancer Research, Frederick, Maryland 21702 USA; 2 Molecular Imaging Program, Center for Cancer Research, National Cancer Institute, Bethesda, USA; 3 Department of Radiology, The Ohio State University Wexner Medical Center, Columbus, OH 43210 USA; 4 Positron Emission Tomography Department, Warren G. Magnuson Clinical Center, National Institutes of Health, Bethesda, USA

**Keywords:** PET/CT imaging, Lesion detection, Human observer study, Signal to noise

## Abstract

**Background:**

A phantom in combination with an imaging protocol was developed to measure the limit of small lesion detection on different PET systems. Seven small spheres with inner diameters ranging from 3.95 up to 15.43 mm were imaged in a Jaszczak ECT Phantom, in air, in a cold background, and with sphere to background contrast ratios of 15:1 down to 1.88:1. The imaging times varied from 1 to 16 min. The imaging protocol was performed on the Gemini TF and Vereos by Philips, the mCT and HRRT by Siemens, and the Discovery 710 by General Electric. For each scanning condition, the images were reconstructed with image voxel sizes of 1 to 4 mm cubic voxels. The reconstruction method used for each system was the one recommended by the manufacture to achieve best small image lesion detection results. A human observer study was performed to determine the smallest observable sphere for each scanning condition.

**Results:**

All systems were able to image the smallest sphere of 3.95 mm inner diameter at the 15 to 1 signal to background ratio when imaged for 16 min. For a typical whole body per bed position scan time of 2 to 4 min, the smallest imaged sphere varied between 4.95 and 6.23 mm at the 15:1 contrast ratio and 12.43 and 15.43 mm at a contrast ratio of 1.88:1. In general, all systems were consistent with the Rose criteria when determining lesion detectability.

**Conclusions:**

Besides demonstrating that the current state of the art clinical PET/CT systems have the same lesion detection ability, the study demonstrates how sensitive scan time can be to detecting small lesions which have a relatively small contrast uptake in the range of just 2:1. This should help guide imaging protocols to use longer scan times over regions of the subject in which small lesions are suspect.

**Electronic supplementary material:**

The online version of this article (doi:10.1186/s40658-017-0179-2) contains supplementary material, which is available to authorized users.

## Background

In the field of PET system development, there is a continuous effort in improving the performance of the next generation system. The technologies adopted and refined in PET systems address basic imaging parameters such as resolution, sensitivity, and aperture (the FOV both radially and axially) which affect the overall quality of the resulting PET image. Extensive research has gone into crystal design, time of flight technology, image reconstruction algorithms, and high speed data acquisition systems, to name a few areas of recent progress. A good reference is the book titled “Positron Emission Tomography” published by Springer [1] which covers all these aspects of the art and science of PET system design.

One of the great advantages of PET system technology is the ability to measure absolute radionuclide activity distributed throughout the field of view of the system. Therefore, besides improving image quality, efforts need to be directed towards image quantification or increasing the accuracy of radionuclide activity measurement. The goal is to measure with greatest accuracy the activity concentration of a radionuclide-labeled agent which has been injected into a human or non-human subject being scanned.

One side field of PET system or PET imaging assessment is that of lesion detection. The ability for a PET system to detect lesions is unique in that it brings to bear all the aspects of a PET system’s features (sensitivity, resolution, reconstruction algorithms) to be able to detect small and/or dim lesions. A lot of effort has gone into the field studying the ability for a PET system to image lesions. These studies have focused on demonstrating improvements in either PET scan design or new image reconstruction techniques using improvements in lesion detection as the metric to assess success in the study at hand. Typically, these studies are performed on a single system and report the improvement attained in lesion detection by introducing a new imaging technology or improvements to reconstruction techniques. For example, how does the use of TOF improve lesion detection as compared to non-TOF. The same question is asked regarding the use of OSEM image reconstruction algorithm versus FBP. Surti, Schaefferkeotter, Larizen, Kadrams, El Fakhri, and Karp are some names of lead authors who have published in this field of study [2–12].

The breadth of research published on lesion detection in PET imaging highlights the importance of this topic and the interest within the molecular imaging oncology field. Being able to detect and measure lesions at their earliest stages of development plays a key role in the treatment staging of cancer patients.

The goal of this study was to assess a PET system’s limit of lesion detection. This is in contrast as to whether a new technology is being validated by measuring the improvement of lesion detection which is the main focus of the publications cited above. The NEMA NU2 PET system performance tests stop short of performing a task which measures the limit of lesion detection. The NEMA NU2 test does provide a lesion imaging test in which hot spheres of different diameters are imaged in a warm background of a large volume phantom with a contrast ratio of either 8:1 or 4:1. All current clinical whole-body PET/CT systems are able to resolve the smallest sphere, thus leaving the main question to be how accurately the system can measure the activity concentration of the smallest sphere. This gives rise to using the contrast recovery coefficient as the metric to compare the imaging capability between systems.

This study attempts to take the NEMA NU2 image quality test to the next level by reducing the size of the spheres, increasing the number of sphere to background contrast settings, changing the scan times, and changing the reconstructed image voxel size to determine under what scanning conditions can a lesion of a certain size be positively observed. With this data set, one should be able to answer the following question. For a lesion of a given size, a tracer uptake to background of a given contrast reconstructed to a given image voxel size, will the lesion be positively observed?

Furthermore, the signal to noise ratio (SNR) for each sphere under all scanning conditions and image reconstructions will be measured and correlated with the lesion observability of the human observer study. The purpose is to correlate the human observer study with the measured parameter SNR to validate the Rose criterion [13].

This article documents the method and results of this lesion detection limit study performed on the following systems; the Gemini TF [14] and Vereos [15] (Philips Medical Systems, Cleveland OH), the Discovery 710 [16] (General Electric Health Care, Waukesha WI) and the Biograph mCT [17], and HRRT [18] (Siemens Health Care, Knoxville TN).

## Methods

### Phantom preparation

We combined four small spheres from a set designed for pre-clinical scanners (ECT/MI-HS/SET4, Data Spectrum Durham, NC) with the three smallest hollow spheres from a set made for clinical scanners (ECT/HS/SET6, Data Spectrum Durham, NC) for a total of seven spheres with inner diameters (and volumes) ranging from 3.95 mm (31 μL) to 15.43 mm (2000 μL). The dimensions of these spheres were taken from the manufacturer’s documentation and are listed in Table 1. These seven spheres were filled with an ^18^F solution and placed inside the Data Spectrum Jaszczak ECT Phantom and imaged under six different imaging conditions. First, the phantom was scanned in air, with no water in the phantom main chamber. Then in cold background, with the main phantom chamber filled with non-radioactive water and finally with four sphere to background contrast ratios of 15:1, 7.5:1, 3.75:1, and 1.88:1.

**Table 1:** Table of sphere dimensions

Inner diameter (mm)	Inner volume (μL)
3.95	31
4.95	63
6.23	125
7.87	250
9.89	500
12.43	1000
15.43	2000

The ^18^F solution concentration used to fill the spheres and background was adjusted such that for the 3.75:1 contrast ratio scan; the background ^18^F solution concentration would be 143 nCi/mL or 5.29 kBq/mL at the start of the PET scan. This was done to match the background activity concentration specified by the NEMA NU2 image quality test. The background activity concentration was prepared using several syringes with a known amount of ^18^F activity. After each scan, the solution in each syringe was sequentially injected into the background volume of the phantom, thus changing the sphere to background ratio from the initial zero background activity to a ratio of 15 to 1, 7.5 to 1, 3.75 to 1, and finally, 1.88 to 1, with each new injection of activity from the prepared syringes. A detailed phantom preparation and imaging SOP is included in the Additional file 1 section.

For each of the six phantom preparations, the phantom was scanned for a total of 16 min. From the 16 min of scanning data, images were reconstructed for the following scan times: 1, 2, 4, 8, and 16 min. For each image reconstruction time, images were reconstructed to a target image voxel size of 1 × 1 × 1 mm (1 mm images), 2 × 2 × 2 mm (2 mm images), and 4 × 4 × 4 mm (4 mm images). Due to the voxel size constraint imposed by the reconstruction software for each system, when the target image voxel dimension was not possible, the closest one to the target dimension was selected. The actual image voxel size for each of the systems is shown in Table 2. One should note the large discrepancy in the target image voxel dimension of the Discovery 710. Due to the manner in which the reconstruction software was written, the slice thickness cannot be changed from 3.27 mm. Therefore, for the Discovery 710, all *Z* dimensions are the same across all target voxel volumes. The PET images were generated using the manufacturer’s recommended reconstruction settings. These settings are tabulated in Table 3.

**Table 2:** Table of system image voxel dimensions. Note: mCT 1.07x1.07x1 voxel volume is achieved through interpolation

Scanner		Target Voxel Size (mm)	
	1x1x1	2 × 2 × 2	4 × 4 × 4
Gemini TF	NA	2 × 2 × 2	4 × 4 × 4
Vereos	1 × 1 × 1	2 × 2 × 2	4 × 4 × 4
Discovery 710	1 × 1 × 3.27	2 × 2 × 3.27	4 × 4 × 3.27
mCT	1.07 × 1.07 × 1	1.99 × 1.99 × 2	4.07 × 4.07 × 4
HRRT	1 × 1 × 1	2 × 2 × 2	4 × 4 × 4

**Table 3:** Reconstruction options used for each scanner

Scanner	Reconstruction Parameters
Gemini TF	BLOB-OS-TOF, 33 subsets, 3 iterations, TOF kernel width 14.1 cm, roll off 0.25
Vereos	3D iterative, 29 subsets, 3 iterations, TOF, PSF regularization parameter 6.0, Gaussian post-filter with 4.1-cm kernel.
Discovery 710	TOF-OSEM, 24 subsets, 5 iterations, PSF resolutionrecovery, Post-filtered with 2-mm Gaussian in transaxial plane.
mCT	PSF + TOF OSEM, 21 subsets, 3 iterations, no postfiltering
HRRT	MOLAR [24], MAP-TR [25]

In order to monitor the signal to background concentration ratio for each scan, the solution used to fill the spheres was sampled three times by pipetting 500 μL of the solution into three separate vials. When the phantom was prepared for scanning at the various contrast ratios, the background volume of the phantom was sampled using a pipette to extract 500 μL of the background volume and placed in vials. After the scanning was done, the radioactivity in the vials was counted in a Perkin Elmer gamma counter providing an independent measure of the signal to background contrast ratios.

### Human observer study

In order to quantify lesion detectability, a human observer study was conducted. Two nuclear medicine physicians and one nuclear medicine technologist were recruited to view each image set and determined which of the 7 spheres they could observe. The observers were given a choice of three observation types to score the lesions with a value of 2, 1, or 0 for lesions which were definitely observed, lesion observed but similar to other noise in the image, and not observed, respectively. Refer to Table 4 for a more detailed description of the lesion scoring scheme.

**Table 4:** Lesion detection scoring table

Score	Definition
2	If the sphere was a lesion, would the observation of the sphere affect the course of treatment of the fictional subject
1	If the sphere was observable but could be similar to other noisy spots in the image and would not qualify as a lesion which would affect the course of the fictional patient’s treatment.
0	If the sphere could not be observed.

Each observer viewed the phantom images using the MIM display software system version 6.5 (MIM Software Inc, Cleveland OH, http://mimsoftware.com). The images were loaded on the viewing station in the three standard views of axial, sagittal, and coronal. The observer was allowed to change the contrast scale, zoom, and pan the images to best tune the images to score the spheres.

Each observer scanned all the image data sets which covered the five systems, four contrast to background settings, five scan times, and three image voxel sizes. All seven spheres for each image data set were scored and recorded for each observer in a database.

The observation scores recorded by the three human observers were aggregated to generate a detectability score of “sphere observed,” “sphere not observed,” or “sphere neither observed nor not observed.” If all three observers scored the sphere with an observation score of 2 or two observers scored the sphere with an observation score of 2 and the third observer scored it with a value of 1, then the sphere was scored as “observed.” If all three observers gave the sphere an observation score of 0 or two observers gave the sphere a score of 0 and the third observer gave it a score of 1, then the sphere was scored as “not observed.” All other combinations of the three observation scores scored the sphere as “neither observed nor not observed.”

### Signal to noise ratio measurement

The signal to noise was measured for each sphere in all images generated in this study. The definition of the signal to noise used in this study is shown in the following equation: [19]

1 $$ \mathrm{S}\mathrm{N}\mathrm{R} = \frac{\left|\overline{s}-\overline{b}\right|}{\sigma_b} $$

where $\overline{s}$ is the mean signal value, $\overline{b}$ is the mean background value, and *σ*_*b*_ is the standard deviation of the background.

The images were processed using MIM image display system to measure the spatial coordinates of each of the spheres for all scans and in-house software algorithms written in C++ were developed using root (CERN, Geneva Switzerland, http://root.cern.ch) to extract the SNR measurements for all spheres.

The challenge with respect to the data analysis of this study is to know the locations of each of the seven spheres within each image. Because the scanning conditions were varied to such extremes, there are image data sets in which none of the spheres can be observed, and thus determining the location of the un-observable spheres within the background is difficult. To solve this problem, the scan in which the spheres were scanned in air was used to determine the relative location of the spheres within the Jaszczak phantom. Then, through a series of image registration steps, the locations of the spheres scanned in air were mapped to the spheres imaged in the four different contrast ratio scans. The details of the registration process can be found in the Additional file 1.

To measure the signal for each sphere, a spherical contour was created which has the same inner diameter as the sphere at the center of the coordinates measured in the previous step. Within this contour, the maximum voxel value was recorded. Next, all the voxels which had a value of 80% of the maximum voxel value or greater were counted and recorded. Of this set of voxels which had values greater than 80% of the maximum, the mean was calculated and recorded.

To measure the warm background, an annulus region, 3 cm thick along the *z*-axis of the FOV, with a 3 cm inner diameter and 7 cm outer diameter in the transaxial plane, was defined and centered at the coordinates for each sphere. The mean and standard deviation of the background activity within this annulus region were measured and recorded.

The signal mean, background mean, and standard deviation were used to calculate the signal to noise ratio defined in Eq. 1.

### MIP image generation and visual contrast scaling procedure

In order to display the images generated in this study for visual comparison between scanning conditions and across the systems, MIP images were generated with their gray scale set to normalize the noise across all images.

To generate the MIP images, the lowest and highest slices were determined along the patient bed (*Z*) axis which contained all seven spheres. Next, a MIP images were formed using the lowest-2 slice through the highest +2 slice. The MIP image will then show the sphere data for all seven spheres in one two-dimensional slice. This procedure of finding the lowest and highest slices was necessary since the spheres do not all line up in a single plane. Also, the phantom was positioned manually on the patient bed and there was enough left or right tilt to move the smallest sphere outside the slice containing the center sphere. The phantom was positioned manually on the patient bed of the clinical PET/CT systems used in the study. Careful attention was paid to ensure the phantom was positioned rectilinearly aided by the laser positioning system of the system. But even so, there was a slight left/right tilt detected once the sphere location in the images was closely inspected.

Next, the mean and standard deviation on a pixel by pixel basis was measured in the area of the MIP image which has only the background data inside the phantom. The area of the MIP image where the spheres were located was excluded. The gray scale was then set such that white (gray scale 0 or minimum scale) was set at −10 standard deviations from the mean background and black (gray scale 256 or maximum scale) is set to +10 standard deviations above mean background. In this way, the visual gray scale of the images is normalized to the noise in the background. This scaling is important when visualizing the smallest lesion in imaging conditions in which the lesions are almost not detectable and allowing one to compare these hard to detect spheres across imaging conditions and systems.

## Results

**Fig. 1:**
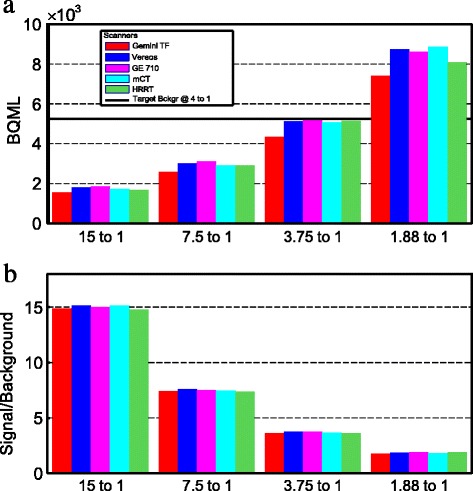
Phantom preparation conditions for the warm background scans. The activity was measured using the PET imaging data and the sphere to background contrast was measured using the pipetted samples. **a** Measured background activity concentration. **b** Achieved sphere to background activity contrast

This study generated a total of 280 images based on images from five systems. This includes all variations of signal to background activity preparations, scan times, and voxel size reconstructions. Furthermore, adding in the seven spheres from each image, we have multiples of 1960 possible data points to present. To simplify the presentation of the data, only a representative sample is depicted here. But for completeness, all 280 images and all data values derived from the study are found in the Additional file 1. Figure 1 contains two bar graphs. The top graph shows the measured background activity measured from the PET image data at the start of the scan for each of the contrast preparations of 15:1, 7.5:1, 3.75:1, and 1.88:1. The bottom graph shows the contrast ratios achieved for each of the target contrast preparation using the pipetted samples extracted from the spheres and from the background volumes.

Table 5 tabulates the results of the human observer study determining the smallest visible sphere in microliters for all the scanning conditions for each of the five systems studied. Each column represents a different scan time in minutes going from 16, 8, 4, 2, and 1 min from left to right. The entry NA means no images were available for that scanning condition and the entry NSV stands for no sphere visible. The results from the human observer study show that all systems were able to image the smallest microsphere of 3.95 mm inner diameter at the 15 to 1 signal to background ratio when imaged for 16 min. For a typical whole body per bed position scan time of 2 to 4 min, the smallest imaged sphere varied between 4.95 and 6.23 mm at the 15:1 contrast ratio and 12.43 and 15.43 mm at a contrast ratio of 1.88:1.

**Table 5:** Results of the human observer study

Sig to noise	Scanner	1 × 1 × 1 mm voxel					2 × 2 × 2 mm voxel					4 × 4 × 4 mm voxel				
		16 min	8	4	2	1	16	8	4	2	1	16	8	4	2	1
15:1	Gemini-TF	NA	NA	NA	NA	NA	31	31	63	63	63	63	125	250	125	250
	Vereos	31	31	31	63	125	31	31	63	63	125	31	63	63	63	125
	710	31	63	63	63	125	31	63	63	63	125	31	63	63	63	125
	mCT	31	63	63	63	63	31	31	63	63	125	31	63	63	63	125
	HRRT	63	63	125	250	500	63	63	125	250	500	63	125	125	500	500
7.5:1	Gemini-TF	NA	NA	NA	NA	NA	125	125	125	125	250	125	125	250	250	500
	Vereos	31	63	125	125	250	31	63	125	125	250	63	63	125	125	250
	710	63	63	63	250	250	63	63	125	250	250	63	63	125	250	250
	mCT	63	63	125	125	250	63	63	125	125	250	63	63	125	125	125
	HRRT	125	125	500	500	500	125	125	250	500	500	125	125	250	500	500
3.75:1	Gemini-TF	NA	NA	NA	NA	NA	250	250	250	500	500	250	500	500	500	500
	Vereos	125	125	250	250	500	125	125	250	250	250	125	250	250	250	250
	710	125	125	250	500	1000	125	125	250	500	500	125	125	250	250	500
	mCT	125	250	250	500	500	125	250	250	500	500	125	250	250	250	500
	HRRT	250	500	1000	1000	NSV	250	500	1000	1000	NSV	500	500	1000	1000	NSV
1.85:1	Gemini-TF	NA	NA	NA	NA	NA	500	1000	2000	2000	NSV	1000	2000	2000	2000	2000
	Vereos	500	500	500	1000	2000	500	1000	1000	1000	2000	500	1000	1000	1000	2000
	710	500	1000	2000	1000	NSV	500	500	2000	NSV	NSV	500	500	1000	1000	NSV
	mCT	500	1000	1000	1000	2000	500	1000	1000	1000	2000	500	1000	1000	1000	2000
	HRRT	1000	NSV	NSV	NSV	NSV	2000	2000	NSV	NSV	NSV	2000	NSV	NSV	NSV	NSV

Tabulated are the volumes in units of microliters of the smallest observed sphere for each imaging condition of signal to noise, scan time, and image voxel size for each of the file PET systems. NA or not applicable is used when no data for that setting was recorded and NSV or no sphere visible is used when none of the spheres were visible.

Table 6 tabulates the SNR measured for a subset of the scans. The purpose of the table is to give one a comparison of SNR values across the five systems which participated in this study. The data for the 7.5:1 contrast ratio scan and the five smallest spheres are presented therein. A full compilation of the SNR values can be found in the Additional file 1.

**Table 6:** Signal to noise ratio summary for 7.5:1 contrast sphere

Scan time	Scanner	1 × 1 × 1 mm voxel					2 × 2 × 2 mm voxel					4 × 4 × 4 mm voxel				
		500 μL	250	125	63	31	500	250	125	63	31	500	250	125	63	31
16 min	Gemini TF	NA	NA	NA	NA	NA	15.1	12.4	9.8	5.4	5.6	78.5	32.9	18.5	5.6	0.6
	Vereos	18.4	15.9	12.9	8.1	9.3	15.3	12.9	10.8	10.3	8.4	82.5	46.8	23.5	11.1	7.1
	710	20.1	20.3	15.5	8.9	2.9	69.5	21.2	9.7	10.0	2.8	73.8	58.2	21.5	9.1	1.1
	mCT	19.0	17.3	12.9	10.3	6.8	12.2	15.4	11.4	8.1	4.7	89.2	51.1	25.0	9.9	4.2
	HRRT	18.0	15.9	12.6	10.9	4.4	10.5	13.8	10.2	7.7	1.9	32.5	20.1	11.1	3.9	0.0
8 min	Gemini TF	NA	NA	NA	NA	NA	15.7	12.0	8.9	4.4	2.3	28.7	14.2	10.3	2.3	1.7
	Vereos	18.0	15.4	12.3	6.9	7.1	15.8	13.0	10.1	6.7	5.8	57.7	26.4	14.6	8.6	4.7
	710	16.9	18.6	13.9	7.7	1.4	73.2	17.7	18.4	6.9	1.0	47.3	42.4	12.2	6.1	0.2
	mCT	19.0	16.3	13.4	9.5	3.6	12.7	15.1	11.5	7.2	3.4	67.7	40.8	20.4	8.8	2.4
	HRRT	17.7	15.1	10.1	7.1	3.6	14.9	15.2	10.0	5.3	1.6	21.5	15.3	9.2	2.6	1.3
4 min	Gemini TF	NA	NA	NA	NA	NA	13.8	10.3	7.7	3.5	2.5	22.9	9.6	5.3	2.9	1.3
	Vereos	17.4	14.3	10.6	5.9	6.0	14.4	12.9	9.3	4.0	4.9	38.2	22.1	10.3	5.6	2.4
	710	17.2	14.6	14.0	8.4	4.8	11.5	12.0	30.7	8.5	4.5	39.7	27.1	12.2	8.4	1.7
	mCT	18.4	16.6	10.1	6.5	3.7	16.6	12.9	9.5	5.1	2.9	55.6	28.7	10.8	4.3	1.7
	HRRT	16.9	14.9	11.3	5.5	2.7	12.5	31.1	20.5	4.1	1.5	14.3	8.5	6.8	3.6	0.8
2 min	Gemini TF	NA	NA	NA	NA	NA	11.7	13.8	8.4	3.4	1.5	14.8	8.2	4.3	3.2	0.5
	Vereos	16.2	13.6	7.0	3.8	5.5	14.8	12.7	6.6	2.2	4.2	28.6	13.9	10.2	3.8	1.3
	710	14.4	14.9	8.8	4.9	1.5	58.8	17.2	6.8	4.5	0.0	27.9	19.1	6.3	0.9	0.0
	mCT	18.4	16.5	10.8	5.1	6.9	19.6	13.0	11.1	3.7	6.9	32.4	17.7	10.4	2.6	5.1
	HRRT	15.3	14.5	11.0	3.3	2.1	12.8	13.4	8.5	2.4	0.2	12.7	9.4	5.6	0.5	0.8
1 min	Gemini TF	NA	NA	NA	NA	NA	13.0	11.0	4.7	3.0	0.9	10.2	4.3	2.3	0.2	0.5
	Vereos	14.5	11.8	8.6	3.8	4.2	13.4	12.0	7.8	1.9	2.9	22.9	11.3	6.3	4.0	1.1
	710	14.8	13.3	10.7	2.0	2.8	14.0	13.5	7.2	1.6	1.1	18.8	20.2	10.9	1.8	0.5
	mCT	17.2	11.4	9.3	5.3	1.2	12.4	10.2	7.4	4.3	0.5	25.8	11.9	8.3	2.7	0.3
	HRRT	13.9	14.9	7.7	3.9	9.7	9.4	12.1	7.3	1.4	3.2	11.1	3.9	1.6	0.5	2.2

**Fig. 2:**
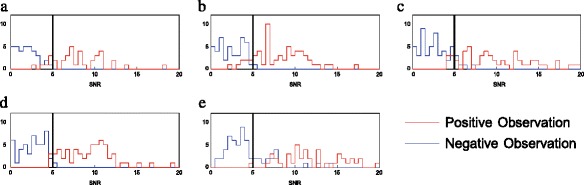
Histograms of the SNR of the positively observed spheres in *red* and positively non-observed spheres in *blue*. The *black vertical line* at 5 marks the Rose criteria threshold of observability. **a** Gemini TF. **b** Vereos. **c** 710. **d** mCT. **e** HRRT

With the tabulated human observer data and the SNR values for each sphere, one can correlate minimum lesion detectability as determined by a human observer with the SNR measured for each sphere. To examine this correlation, a histogram of SNR for minimally observed spheres and for non-observed spheres is present, one for each system in Fig. 2. The two sets of spheres, the observable ones and the non-observable ones, are shown to be separated from each other with the observable spheres in general having a SNR greater than 5 and the non-observable spheres having a SNR less than 5. This agrees with the Rose criterion named after Albert Rose, who studied object observability back in the 1970s. He established an observability criterion such that for a small object to be observed, it had to have a SNR greater than 5 [13].

**Fig. 3:**
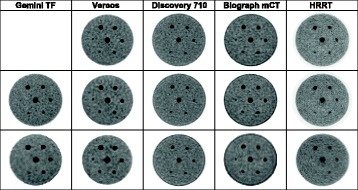
Scanner comparison, 7.5 to 1 contrast ratio. Top row contains 1-mm voxel reconstructions, middle row is 2 mm voxel reconstructions and last row is 4-mm voxel reconstructions. All images are 16-min scans

**Fig. 4:**
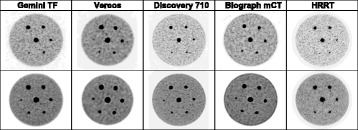
Scanner comparison, 15 to 1 contrast ratio. Top row is 2-min acquisitions, bottom row is 16-min acquisitions. All images were reconstructed to 2 mm voxel size

The final data presentation summary is a select set of images of the spheres which can be found in Figs. 3 and 4. Figure 3 gives a side by side visual comparison of the 7.5:1 signal to background preparation for the five systems. All images represent a 16-min acquisition and reconstructed to the three target voxel sizes of 1 × 1 × 1 mm, 2 × 2 × 2 mm, and 4 × 4 × 4 mm. The images in Fig. 4 show images of the phantom for a 15:1 signal to background phantom preparation. The top row is 2-min scans and the bottom row is 16-min scans.

## Discussion

The goal of the study was to survey the imaging parameter space of lesion to background contrast ratios, scan time, image voxel size, and lesion volume in order to determine a system’s limit of detecting small lesions. The idea is to measure this important clinical capability which is not addressed by conventional phantoms. Typical “resolution” phantoms rely on high contrast objects whereas the “real world” task is to detect relatively lower contrast objects. Keep in mind the NEMA NU2 image quality test, the smallest hot sphere has an inner diameter of 10 mm. This sphere size is too large to measure the limits of detection.

For the comparison of minimum lesion detectability across systems, it was vital to ensure that the phantom preparation was consistent so that each system imaged the phantom at the same signal to background concentration ratio and absolute activity in the field of view at the start of each scan. The data presented in Fig. 1 establish that the phantom was prepared consistently for each system. Note that the absolute activity for the Philips Gemini TF is lower by 14% on average between each of the 4 concentration ratios. This small deviation of lower absolute activity should have a minimal affect by slightly reducing the ability to image the smallest lesion. The difference is considered to be negligible since the concentration ratios were all within 2% of each other as seen in the bottom plot of Fig. 1.

With the phantom data acquired on the five systems, the next step was to reconstruct the images in a manner which would be best suited to compare the system’s ability to image small lesions. Since PET reconstruction algorithms are complex and implemented in a unique way on each system, it was left to each system operator to use whatever setting and algorithms were available to generate what the manufacturer would consider the best reconstruction settings to generate the best quality images. Therefore, the number of iterations, subsets, and postfiltering parameters which can play a role in adjusting the image quality of the system was not specified. See Table 3 which tabulates the reconstructions settings used for each system. The only specification for image generation was the voxel size of 1 × 1 × 1 mm, 2 × 2 × 2 mm, and 4 × 4 × 4 mm.

This brings us to the main result of the study, which is to determine the minimum lesion size which is observable under the different scanning conditions explored by this study. The main results are found in Table 5. This table lists which was the smallest detected sphere from the human observer study for each scan and image reconstruction condition and elucidates some expected results. The longer one scans, the smaller the lesion that can be observed. The higher the signal to background contrast ratio, the smaller the lesion can be observed. A more subtle effect is the image voxel size. One can pick out some cases in which for the same scanning condition of scan time and contrast ratio, changing the voxel size, will allow one to observe a smaller lesion. For example, the Vereos system demonstrates that for a 15:1 signal to background contrast ratio and a scan time of 8 min; it can detect the 31 μL sphere with a 2 × 2 × 2 mm voxel image which cannot be done on a 4 × 4 × 4 mm voxel image. Searching through Table 5, one can find other examples of improved lesion detection when an image is reconstructed using a smaller voxel size. This effect of improving the lesion detectability of the system by reconstructing the images with a smaller voxel size has also been published by Morey et al. [20]. Kadrmas et al. [21] have published data showing improving lesion detection as one increases scan time.

The change in lesion detection for different voxel size reconstructions is also a case study in partial volume effects. Soret et al. [22] have a very nice discussion on how pixel or voxel size partial volume effects for small lesions affect the reconstructed lesion. Mainly for small lesions under two to three full-width at half maximum resolution of the scanner, the larger the full-width at half maximum resolution of the system, the impact on lesion detection is greater the larger the pixel or voxel size is, with the larger voxels degrading the lesion due to spill-out effects. Due to the warm background, there are larger spill-in effects as well. The data set acquired for this study will provide for a good analysis of partial volume effects, especially since the study focuses on small lesions which push the resolution limits of the systems. The results from a full study on partial volume effects will be published in a separate manuscript.

If we were to summarily state an overall result for this study, this would be that for a typical 2 to 4 min scan time per bed position, one can detect a lesion as small as 0.5 cm in diameter if the contrast to background ratio is 15:1 or better. For lesions with a contrast to background ratio of at least 2:1, the smallest lesion that one can detect will be 1.5 cm in diameter.

Table 6 and the accompanying Table 5 list the SNR values for a subset of images and give a more fine-grained measure of how well the system was able to resolve a lesion. But one should be careful not to rely on a few units of SNR to claim one system can better resolve a lesion scanned under a given condition than another.

In a not very surprising confirmation of the Rose criterion, the histograms shown in Fig. 2 show how observable lesions and non-observable lesions fall on either side of the dark line drawn at a SNR of 5.

The final set of data presented are the actual images of the phantoms so one can gauge via visual inspection the detectability of the spheres for a few selected imaging conditions. Figure 3 displays the maximum intensity projection (MIP) images for the slices which contain any of the seven spheres. The goal is to limit the number of slices over which one applies the MIP algorithm to only those which have sphere data and thus reduce the overall noise in the warm background volume of the phantom. Refer to the “Methods” section where a more detailed explanation of how the MIP images were prepared for display in Figs. 3 and 4. The images are for each system for the 7.5 to 1 signal to background contrast ratio. The top row is for 1 × 1 × 1 mm voxel images, the second row for 2 × 2 × 2 mm voxel images, and the last row for 4 × 4 × 4 mm voxel images. In this case, one can see how the voxel size can affect lesion detectability due to voxel partial volume effects as discussed earlier. Notice in the HRRT data, how the 63 μL sphere at the 7 o’clock position is clearly visible in the top row (1 × 1 × 1 mm voxel image) and is not present in the bottom row (4 × 4 × 4 mm voxel image).

Figure 4 shows images for different scan times. These are the 15 to 1 signal to background concentration ratio images. The top row is the 1-min scan time images and the bottom row is the 16-min scan time images. All images were reconstructed to 2 mm voxel size. In most cases, the smallest lesion at the 9 o’clock location cannot be resolved in the 1-min acquisition time images while it can be resolved in the 16-min acquisition time images. The exception is the HRRT scanner in which the smallest lesion was not detected at all.

To complete the discussion, the shortcomings of the study design should be mentioned. The first one is that the spheres are all in the same location removing the element of random placement thus giving the human observer a known location to search for the lesion. The other disadvantage is that the spheres have relatively thick plastic shells which will decrease the actual observability. This has been shown by Berthon et al. [23] and should be taken into account. Another drawback of the study is that the diameter of the Jazczak QC PET phantom used in this study is not comparable to a human torso. Studies have shown that lesion detectability varies greatly as a function of subject BMI as noted by Karp et al. [6]. Therefore, one should not take the results listed in Table 5 as absolute but perhaps as a lower limit.

For the reader who wishes to do a full examination of all the image data generated by this study, all the images can be found in the Additional file 1 section.

## Conclusions

The publication of the data presented herein is the result of a comprehensive study of PET/CT systems undertaken by the NCI Molecular Imaging Program. The goal was to assess the current state of the art of PET/CT systems. Typically, one would perform a literature search and tabulate the NEMA NU2 measurements for each PET/CT system. Here, we took one step further and performed scans in all systems available using a single phantom that would best challenge the PET/CT system design. The study of how small a lesion a PET/CT system could image was chosen as the main outcome since this parameter is one that is clinically relevant in cancer detection.

## Additional file

Additional file 1: Minimum lesion detectability as a measure of PET scanner performance. (PDF 21569 kb)
