# Supplementary material for: Minimum lesion detectability as a measure of PET system performance
Source: EJNMMI Phys. 2017 Mar 4;4:13. doi: 10.1186/s40658-017-0179-2 (PMC5337231; doi:10.1186/s40658-017-0179-2)
Supplement: Additional file 1: — Minimum lesion detectability as a measure of PET scanner performance. (PDF 21569 kb) [file 40658_2017_179_MOESM1_ESM.pdf]

Noname manuscript No.
(will be inserted by the editor)

Minimum Lesion Detectability as a Measure of PET Scanner Performance

Supplemental Material

Stephen Adler · Jurgen Seidel · Peter Choyke · Michael V Knopp · Katherine Binzel · Jun Zhang · Craig Barker · Shielah Conant · Roberto Maass-Moreno

Received: date / Accepted: date

1 Supplemental Material

The following pages contain all the supplemental material generated from this scanner comparison study.

The first section contains the phantom preparation and scanning standard operating procedure (SOP) document. This is included in case someone wishes to replicate the scanning conditions used for this study.

The next section details the registration procedure used to register the PET images of the spheres scanned in air to the PET images scanned in a warm background using the MIM software image display system.

This is followed by a set of tables tabulating all the SNR measurements.

The supplemental material finishes with a display of every image acquired for the study. For each page, one has a matrix of images with each row representing the different activity concentration ratios and each column the acquisition time. The top to bottom row ranging from 15 to 1, 7.5 to 1, 3.75 to 1 and 1.88 to 1 concentration ratios. The left to right columns, the acquisition times are 1 minute, 2 minute, 4 minute, 8 minute and the right most being 16 minute acquisition times. Each page has images for a different image voxel size and scanner. The images are maximum intensity projects of the slices which contained the spheres and the gray scale is normalized to the noise in the warm background as described in the main text of the manuscript.

1.1 Phantom Filling and Scanning SOP

1.1.1 Introduction

This Standard Operation Procedure describes the procedure by which the phantom is prepared and scanned to measure a PET scanners minimum lesion detectability.

Address(es) of author(s) should be given

1.1.2 Minimum Lesion Detectability Scan Summary

The goal of the minimum lesion detectability scan is to understand and/or measure the limits of small lesion detection of the scanner. This is achieved by preparing a uniform background volume of 18F activity with hot micro spheres placed within this uniform background volume.

The microspheres vary in inner diameter from 15.43 mm down to 3.95 mm. Key to understanding the small lesion detectability is to adjust the sphere activity to background activity ratio between a signal to background ratio (SUV) of 15, 7.5, 3.75 and 1.875. Furthermore, the acquisition time should be varied between 1 and 16 minutes, and the image voxel size should be varied between $1mm^3$ to $4mm^3$.

The following SOP describes the phantom preparation procedure, the scanning procedure and image reconstruction procedure. When done, the following 45 image data sets generated.

1. 16 to 1 sphere to background image with the following acquisition and reconstruction parameters
   (a) 1, 2, 4, 8 and 16 minute acquisitions each reconstructed in $1mm^3$, $2mm^3$ and $4mm^3$ voxels
2. 8 to 1, 4 to 1 and 2 to 1 background images reconstructed as specified for the 16 to 1 images.

1.1.3 Materials and Methods

The following materials are required for the phantom scan procedure

1. Flanged Jaszczak Cylinder Phantom (NOTE: the volume of the Flangless Jaszczak phantom is 94.44% that of the size of the flanged phantom, thus one needs to adjust the weight of the initial dilution volume in step 5 of the phantom filling section accordingly.)
2. Microspheres ranging from 3.95 mm inner diameter to 15.43 mm
3. Micro Hollow Sphere Set (4) (Model ECT/MH-HS/SET4)
4. Three smallest spheres from Hollow Sphere Set (6) (Model ECT/HS/SET6)
5. Five 5cc syringes
6. Scale with accurate 10 Kg range
7. Scale with accurate 1g range
8. Liquid absorption pads
9. 1cc pipet
10. 15 1cc vials
11. 500ml bottle

The method to fill the phantom is as follows.

1. Label and weigh and record the weights of the fifteen 1cc vials using the 1g range scale
   (a) Weights should be recorded to 3 decimal places
2. Place an absorption pad on the scale to protect from spillage
3. Place the 500 ml bottle on the absorption pad on the scale.
4. Zero out the scale
5. Fill the phantom with water until the scale reads 454 grams

6. Prepare the following set of doses (+/- 10%)
   (a) Syringe #1: 628 uCi
   (b) Syringe #2: 628 uCi
   (c) Syringe #3: 1256 uCi
   (d) Syringe #4: 2512 uCi
7. Record the dose and time of the dose measurement. The times of the syringe dose measurements should be within 2 minutes. The mean time will be the Injection Time
8. Empty syringe #1 in 500cc bottle. Screw the top on the bottle and mix well to ensure activity is uniformly distributed in the liquid volume
9. Extract 5ccs of the mix into the 5th 5cc syringe. This will be used later to fill the hollow spheres.
10. Using the pipet, fill three vials labeled #1, #2 and #3 with .5 cc from the solution in the bottle.
11. Fill the hollow spheres with the solution extracted from the phantom.
12. If possible, measure the activity from each sphere in either a well counter or gamma counter.
13. Place the filled hollow spheres in the phantom following the diagram of figure 1.
14. Close the phantom, leaving the main chamber volume empty.
15. Proceed to scan the phantom.
16. Fill the chamber of the phantom with non-radioactive water (cold water).
17. Proceed to scan the phantom.
18. Empty 1000cc of cold water from the phantom. Take the contents of the 500cc bottle which has had the 1st syringe dose mixed in, and mix into the chamber volume of the phantom. Finish filling up the chamber volume with cold water. Mix well.
19. Using the pipet, fill vials #4, #5, #6 with .5cc solution from the chamber of the phantom.
20. Proceed to scan the phantom 90 minutes post syringe measurement time recorded in step #7.
21. Inject Syringe #2, mix, and fill vials #7, #8, #9 with .5cc of background solution.
22. Proceed to scan the phantom, starting the PET scan 30 minutes after the start of the PET scan from the previous PET scan.
23. Repeat with Syringe #3 and #4, filling vials #10, #11, #12 after mixing the background with syringe #3, and fill vials #13, #14, #15 after mixing the background with syringe #4. Space each PET scan 30 minutes apart.

1.1.4 Scanning the phantom

1. Placed phantom on the patient bed and position the plane of the spheres transaxially
2. Adjust the bed height and left/right position of the phantom on the bed to center the circle defined by the spheres in the center of the FOV. The plane defined by the circle should be centered axially as well.
3. Perform a standard low dose CT scan
4. For the phantom with no water in the background changer or just cold water in the background chamber, Perform an 8 minute dynamic PET scan using standard energy window settings.

5. For the phantom with a hot background, perform a 16 minute dynamic PET scan using standard energy window settings

1.1.5 Reconstructing the phantom images

The goal of the image reconstruction is to generate images of different scan times and voxel sizes. There are 5 scan times, these are 1 minute, 2 minute, 4 minute, 8 minute and 16 minute for the scans in which the background had hot activity in it. Scan times of 1, 2, 4 and 8 for the scans with the background changer empty or filled with cold water. The target image voxel sizes are 1x1x1 mm, 2x2x2 mm, 4x4x4mm and the standard voxel size for whole body imaging as defined by the scanner operating software. The following steps will generate the needed images.

1. Reconstruct the images as a dynamic image sequence with the following time frame sequence.
   (a) Two 1 minute time frames, One 2 minute time frame, One 4 minute time frame, One 8 minute time frame for the scans with a hot background.
   (b) Two 1 minute time frames, One 2 minute time frame, One 4 minute time frame, for the scans with the background chamber empty or filled with cold water.
2. Reconstruct the images a one static image set for the whole (8 minutes) 16 minutes of scan time.
3. For each reconstruction defined in steps 1 and 2, generate the following image voxel size.
   (a) Default image voxel size used to reconstruct whole body images.
   (b) If possible generate further reconstructed dynamic image sequences using the following image voxel size. 4x4x4mm, 2x2x2mm, 1x1x1mm
4. For all reconstructions, use the best reconstruction algorithm available to the scanner. (i.e. TOF with PSF etc.)

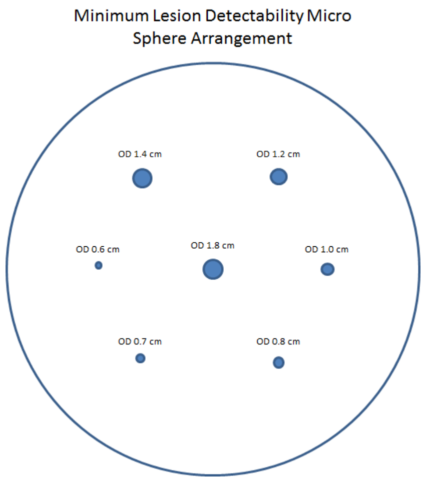

Fig. 1 Sphere arrangement within the Jaszczak phantom

1.2 Sphere Location Measurement Procedure

The specific steps to achieve this registration of spheres scanned in air to spheres scanned in a warm background was achieved using the MIM display software image registration tool.

The following steps were taken to register the spheres scanned in air PET image with the four spheres scanned in warm background PET images.

One starts by loading the PET and CT images for the spheres scanned in air and then drawing a threshold delineated 3D contours on each of the spheres on the PET image with the threshold set to 5% of maximum. Because the PET and CT images are spatially registered, one can transfer the contours from the PET images to the CT images. Next one loads the PET/CT image data set of the spheres scanned in a warm background. Using the MIM image registration tool, one then registers the CT image of the spheres scanned in air with the CT of the spheres scanned in a warm background. This now has the secondary effect of registering the initial contours drawn on the PET images of the spheres scanned in air, to the CT image of the spheres scanned in warm background. Finally one transfers the contours now registered on the CT images of the spheres scanned in the warm background to the accompanying PET images of the spheres scanned in warm background. The final location of the spheres in the warm background PET image is then recorded and stored in a sphere co-ordinate database.

Table 1 Signal to Noise Ratio for the Gemini TF Scanner

Philips Gemini TF													
		1 vox				2 vox				4 vox			
		15:1	7.5:1	3.75:1	1.88:1	15:1	7.5:1	3.75:1	1.88:1	15:1	7.5:1	3.75:1	1.88:1
2000 ul	16 min	NA	NA	NA	NA	21.9	18.3	15.0	7.2	8.8	11.6	9.9	5.5
	8 min	NA	NA	NA	NA	24.2	19.5	13.1	7.5	15.6	12.7	12.5	4.8
	4 min	NA	NA	NA	NA	23.8	18.5	13.4	6.6	15.0	13.0	9.7	5.3
	2 min	NA	NA	NA	NA	24.4	14.9	13.0	6.7	15.8	13.2	9.5	4.5
	1 min	NA	NA	NA	NA	17.6	13.2	8.5	5.4	15.4	11.6	8.7	4.8
1000 ul	16 min	NA	NA	NA	NA	18.9	18.6	13.3	5.0	13.1	22.1	12.4	2.7
	8 min	NA	NA	NA	NA	16.5	17.4	13.0	5.2	14.2	9.4	8.2	2.4
	4 min	NA	NA	NA	NA	19.2	16.4	10.6	5.7	13.5	9.5	9.3	1.8
	2 min	NA	NA	NA	NA	20.5	16.6	10.1	2.3	14.7	15.0	7.3	0.9
	1 min	NA	NA	NA	NA	16.4	13.8	9.3	1.4	16.3	11.1	7.4	1.2
500 ul	16 min	NA	NA	NA	NA	18.3	15.1	10.4	3.6	107.7	78.5	20.7	1.5
	8 min	NA	NA	NA	NA	14.6	15.7	10.1	2.9	57.4	28.7	12.3	0.5
	4 min	NA	NA	NA	NA	16.0	13.8	9.1	2.4	36.2	22.9	10.5	0.2
	2 min	NA	NA	NA	NA	16.0	11.7	8.2	1.9	32.0	14.8	8.4	0.4
	1 min	NA	NA	NA	NA	13.7	13.0	7.4	2.0	19.8	10.2	4.1	1.1
250 ul	16 min	NA	NA	NA	NA	15.4	12.4	7.3	0.3	42.5	32.9	10.4	1.6
	8 min	NA	NA	NA	NA	15.0	12.0	6.5	0.1	32.9	14.2	3.2	2.0
	4 min	NA	NA	NA	NA	12.8	10.3	6.4	0.5	23.9	9.6	2.1	1.1
	2 min	NA	NA	NA	NA	18.8	13.8	5.0	0.8	18.6	8.2	2.2	1.7
	1 min	NA	NA	NA	NA	16.0	11.0	4.3	0.1	12.1	4.3	0.7	0.2
125 ul	16 min	NA	NA	NA	NA	16.5	9.8	4.2	0.8	43.3	18.5	3.0	1.5
	8 min	NA	NA	NA	NA	19.6	8.9	2.9	0.3	13.8	10.3	1.2	1.4
	4 min	NA	NA	NA	NA	14.1	7.7	1.6	0.5	4.2	5.3	0.1	0.0
	2 min	NA	NA	NA	NA	12.4	8.4	1.7	0.5	7.3	4.3	0.4	1.5
	1 min	NA	NA	NA	NA	10.5	4.7	0.1	0.0	3.8	2.3	1.0	1.2
63 ul	16 min	NA	NA	NA	NA	16.9	5.4	0.2	1.7	22.3	5.6	0.4	1.5
	8 min	NA	NA	NA	NA	13.4	4.4	0.1	1.0	7.5	2.3	1.0	1.5
	4 min	NA	NA	NA	NA	10.6	3.5	1.4	0.4	4.1	2.9	0.1	0.1
	2 min	NA	NA	NA	NA	6.9	3.4	1.0	0.9	4.8	3.2	0.6	0.1
	1 min	NA	NA	NA	NA	7.2	3.0	0.0	0.1	2.6	0.2	0.3	0.4
31 ul	16 min	NA	NA	NA	NA	11.0	5.6	0.8	3.5	7.6	0.6	3.1	5.4
	8 min	NA	NA	NA	NA	7.1	2.3	0.2	2.7	3.9	1.7	1.9	3.0
	4 min	NA	NA	NA	NA	6.2	2.5	0.7	1.2	1.3	1.3	1.3	2.7
	2 min	NA	NA	NA	NA	4.2	1.5	0.6	0.7	0.7	0.5	1.2	1.1
	1 min	NA	NA	NA	NA	2.6	0.9	0.6	0.7	4.1	0.5	0.4	0.9

Table 2 Signal to Noise Ratio for the Vereos Scanner

Philips Vereos													
		1 vox				2 vox				4 vox			
		15:1	7.5:1	3.75:1	1.88:1	15:1	7.5:1	3.75:1	1.88:1	15:1	7.5:1	3.75:1	1.88:1
2000 ul	16 min	21.3	19.7	15.8	9.0	21.2	18.0	15.9	9.0	11.9	17.7	11.7	7.1
	8 min	21.9	19.2	15.8	8.7	22.5	17.5	15.6	8.9	15.6	12.3	11.0	7.7
	4 min	22.1	19.0	15.1	8.5	25.1	18.1	16.3	8.8	14.6	12.2	10.8	5.8
	2 min	21.5	19.7	14.5	7.4	23.3	18.0	15.4	8.4	12.9	12.5	10.6	5.9
	1 min	20.2	18.6	12.8	6.8	19.4	17.4	13.5	7.0	13.7	11.8	11.2	6.8
1000 ul	16 min	21.1	19.1	16.4	8.0	18.9	15.7	15.0	6.7	10.9	16.3	10.7	4.4
	8 min	21.1	20.0	15.3	7.7	19.5	15.7	14.9	6.5	12.4	18.3	11.0	4.7
	4 min	21.7	19.2	14.3	7.2	19.1	16.1	14.3	5.7	11.3	17.7	9.3	3.6
	2 min	21.7	18.6	12.6	6.4	18.7	14.3	12.9	5.6	14.5	30.1	10.4	5.1
	1 min	20.0	17.9	11.4	4.4	17.4	12.7	11.9	4.4	13.2	24.1	9.0	3.2
500 ul	16 min	20.9	18.4	13.9	4.1	17.2	15.3	12.5	2.4	146.6	82.5	38.2	3.5
	8 min	20.6	18.0	13.1	3.9	16.7	15.8	11.8	2.5	101.4	57.7	21.5	3.6
	4 min	19.7	17.4	12.2	4.5	16.6	14.4	11.3	2.9	72.8	38.2	18.9	1.1
	2 min	20.0	16.2	12.0	4.7	16.0	14.8	10.8	2.8	54.5	28.6	12.5	2.1
	1 min	18.5	14.5	10.4	4.4	16.1	13.4	10.1	1.7	43.8	22.9	8.0	0.3
250 ul	16 min	19.0	15.9	10.3	2.4	16.3	12.9	9.4	1.4	85.7	46.8	18.6	0.9
	8 min	19.2	15.4	9.7	1.9	14.9	13.0	8.6	0.9	54.6	26.4	13.6	0.5
	4 min	18.5	14.3	9.5	0.9	18.3	12.9	7.5	0.2	37.0	22.1	9.3	0.2
	2 min	17.7	13.6	8.1	1.2	16.9	12.7	7.6	0.2	30.0	13.9	5.5	0.2
	1 min	14.9	11.8	7.0	0.7	13.7	12.0	6.6	0.7	14.0	11.3	5.1	0.4
125 ul	16 min	18.0	12.9	6.0	2.2	12.8	10.8	6.9	2.7	41.4	23.5	8.7	3.5
	8 min	17.7	12.3	5.7	2.7	14.3	10.1	4.5	2.8	26.4	14.6	6.5	1.3
	4 min	15.9	10.6	3.7	1.6	12.3	9.3	2.9	2.9	17.8	10.3	6.2	3.4
	2 min	14.4	7.0	3.9	2.9	11.6	6.6	3.0	3.8	13.1	10.2	3.9	0.5
	1 min	12.7	8.6	4.3	1.3	10.8	7.8	3.2	2.3	10.5	6.3	2.9	1.6
63 ul	16 min	15.5	8.1	2.9	3.4	30.4	10.3	1.5	3.7	26.4	11.1	0.1	3.2
	8 min	13.9	6.9	4.0	3.2	20.5	6.7	2.7	2.7	17.1	8.6	0.4	3.6
	4 min	12.2	5.9	3.7	3.0	14.1	4.0	2.4	2.7	9.9	5.6	1.1	0.8
	2 min	10.0	3.8	3.7	1.5	9.7	2.2	2.2	1.8	10.8	3.8	0.3	0.7
	1 min	8.2	3.8	5.3	0.9	6.6	1.9	3.3	1.4	3.8	4.0	1.5	1.4
31 ul	16 min	11.3	9.3	1.3	4.5	11.7	8.4	0.1	5.1	7.8	7.1	1.1	4.0
	8 min	9.2	7.1	0.4	3.4	10.1	5.8	1.0	2.6	3.8	4.7	1.2	2.7
	4 min	8.3	6.0	1.5	2.3	8.3	4.9	1.7	1.5	4.8	2.4	0.3	1.0
	2 min	5.8	5.5	0.2	2.1	5.2	4.2	1.0	1.4	3.8	1.3	0.7	0.1
	1 min	4.1	4.2	1.6	1.1	3.6	2.9	0.9	0.3	1.0	1.1	1.2	0.1

Table 3 Signal to Noise Ratio for the Discovery 710 Scanner

Discovery 710													
		1 vox				2 vox				4 vox			
		15:1	7.5:1	3.75:1	1.88:1	15:1	7.5:1	3.75:1	1.88:1	15:1	7.5:1	3.75:1	1.88:1
2000 ul	16 min	25.2	21.1	16.1	9.9	23.8	20.9	16.8	9.5	25.8	15.4	14.4	8.8
	8 min	22.4	19.3	16.9	8.6	18.0	16.4	16.4	7.8	21.5	16.8	14.6	8.3
	4 min	21.5	18.8	12.8	9.7	21.0	15.9	12.8	9.0	20.1	14.5	12.5	9.4
	2 min	20.0	15.9	11.9	7.2	20.1	14.8	12.1	6.9	18.3	13.0	9.8	5.6
	1 min	16.9	17.4	13.4	5.4	21.9	15.6	10.9	4.9	13.5	14.3	10.2	4.7
1000 ul	16 min	20.8	18.6	15.6	8.8	21.2	14.1	16.0	7.7	22.2	12.7	9.6	7.2
	8 min	22.3	17.9	13.4	9.1	17.5	18.0	11.8	8.3	18.7	13.1	11.1	7.4
	4 min	21.5	18.3	14.0	5.2	17.9	18.0	12.4	5.0	16.8	16.7	7.4	4.8
	2 min	20.7	18.8	13.3	7.0	18.3	13.1	11.8	6.9	11.8	14.5	19.7	5.3
	1 min	19.0	16.2	12.2	4.2	14.1	13.6	9.2	4.6	33.7	35.0	7.1	3.5
500 ul	16 min	18.4	20.1	13.2	4.8	12.1	69.5	10.1	4.3	140.8	73.8	36.7	4.1
	8 min	18.8	16.9	14.2	5.2	17.1	73.2	15.1	5.2	97.8	47.3	29.5	4.1
	4 min	20.1	17.2	12.2	4.8	12.9	11.5	12.3	4.2	65.4	39.7	13.7	2.6
	2 min	17.9	14.4	11.2	3.6	88.3	58.8	23.5	3.3	46.8	27.9	12.8	1.3
	1 min	19.5	14.8	9.8	2.6	10.8	14.0	8.4	1.9	41.0	18.8	8.9	1.1
250 ul	16 min	16.7	20.3	12.1	2.3	18.5	21.2	26.5	2.3	72.2	58.2	18.6	2.3
	8 min	17.2	18.6	11.3	1.1	31.7	17.7	12.4	1.1	54.1	42.4	13.7	0.4
	4 min	18.7	14.6	8.8	2.8	46.4	12.0	6.8	2.8	35.7	27.1	8.7	2.2
	2 min	14.1	14.9	7.2	2.9	85.7	17.2	8.6	2.9	18.5	19.1	4.5	2.0
	1 min	15.3	13.3	7.1	2.2	16.1	13.5	7.3	2.2	17.7	20.2	4.4	1.7
125 ul	16 min	14.1	15.5	6.7	1.1	36.0	9.7	6.0	0.9	48.0	21.5	8.2	0.4
	8 min	15.0	13.9	6.4	0.5	29.1	18.4	6.3	0.2	34.2	12.2	7.0	0.2
	4 min	15.4	14.0	7.3	0.3	25.0	30.7	8.1	0.1	25.7	12.2	5.6	1.0
	2 min	15.9	8.8	6.0	1.6	46.1	6.8	1.3	0.7	17.8	6.3	0.2	0.6
	1 min	14.0	10.7	1.2	0.4	10.6	7.2	1.0	0.1	12.5	10.9	0.3	1.3
63 ul	16 min	16.3	8.9	4.7	0.4	30.0	10.0	4.8	0.1	28.3	9.1	6.5	0.6
	8 min	15.1	7.7	3.3	0.2	17.4	6.9	2.6	0.4	19.8	6.1	3.0	0.0
	4 min	12.9	8.4	4.9	1.3	12.6	8.5	3.9	1.2	15.7	8.4	5.1	0.3
	2 min	18.7	4.9	2.5	0.5	14.3	4.5	1.4	0.3	14.7	0.9	2.0	0.5
	1 min	7.2	2.0	1.9	0.9	6.0	1.6	1.4	0.5	2.4	1.8	1.8	1.7
31 ul	16 min	10.2	2.9	3.8	0.1	12.1	2.8	2.9	0.3	10.8	1.1	1.1	0.8
	8 min	8.4	1.4	3.5	0.5	7.4	1.0	2.7	0.4	8.2	0.2	1.4	0.5
	4 min	5.3	4.8	1.5	1.0	4.7	4.5	0.9	0.8	2.4	1.7	0.2	0.5
	2 min	6.1	1.5	1.2	0.3	5.5	0.0	1.1	0.3	3.5	0.0	0.1	0.1
	1 min	2.9	2.8	0.5	1.4	1.4	1.1	0.5	1.1	2.1	0.5	0.2	1.2

Table 4 Signal to Noise Ratio for the mCT Scanner

Siemens mCT													
		1 vox				2 vox				4 vox			
		15:1	7.5:1	3.75:1	1.88:1	15:1	7.5:1	3.75:1	1.88:1	15:1	7.5:1	3.75:1	1.88:1
2000 ul	16 min	21.6	24.4	17.8	10.4	23.2	22.1	17.2	9.8	18.4	25.3	20.1	16.6
	8 min	22.7	24.8	19.0	10.2	24.8	22.6	16.5	8.6	25.1	24.4	13.1	11.6
	4 min	23.5	21.8	15.4	8.8	21.0	18.5	14.8	9.0	18.1	16.9	21.2	9.2
	2 min	23.4	20.2	15.1	9.3	18.0	19.0	14.4	8.4	15.7	12.6	13.3	6.9
	1 min	21.6	18.6	15.4	10.6	16.9	13.2	14.7	8.6	22.2	15.6	14.0	15.0
1000 ul	16 min	22.6	20.3	19.0	8.4	21.0	17.9	15.4	7.3	21.2	12.8	10.6	4.5
	8 min	22.9	20.9	16.1	6.9	18.4	17.9	15.7	6.8	26.0	11.2	11.3	4.7
	4 min	22.1	20.0	15.5	7.8	19.3	20.1	13.2	5.9	20.5	24.5	10.7	4.8
	2 min	21.0	20.0	15.5	7.0	23.0	14.0	14.4	6.4	14.0	8.1	18.8	5.6
	1 min	20.4	17.8	11.3	4.7	15.6	15.2	11.3	4.4	14.9	25.9	7.9	4.0
500 ul	16 min	22.1	19.0	16.4	5.1	16.9	12.2	12.0	5.0	236.8	89.2	32.0	4.5
	8 min	19.7	19.0	15.2	5.2	17.6	12.7	11.2	4.6	168.0	67.7	26.7	3.6
	4 min	23.7	18.4	13.8	4.3	14.9	16.6	12.8	3.8	128.1	55.6	15.0	2.0
	2 min	18.4	18.4	13.6	3.3	20.9	19.6	11.0	2.9	90.5	32.4	12.6	1.8
	1 min	20.9	17.2	12.3	2.2	16.0	12.4	9.9	3.0	66.4	25.8	9.9	2.1
250 ul	16 min	17.1	17.3	10.7	0.6	14.6	15.4	8.7	0.1	117.5	51.1	16.5	1.0
	8 min	15.5	16.3	10.7	0.3	15.8	15.1	6.9	0.3	80.3	40.8	11.3	0.2
	4 min	15.9	16.6	9.5	1.9	18.1	12.9	7.6	1.6	71.7	28.7	9.9	1.2
	2 min	17.1	16.5	7.5	0.3	12.9	13.0	6.7	0.8	40.3	17.7	6.4	0.5
	1 min	13.8	11.4	4.0	1.1	15.2	10.2	4.1	0.8	37.9	11.9	3.0	0.7
125 ul	16 min	17.6	12.9	6.0	1.8	53.5	11.4	5.3	0.9	39.4	25.0	7.7	1.2
	8 min	17.2	13.4	5.6	1.1	64.3	11.5	4.4	0.3	27.8	20.4	6.0	0.6
	4 min	17.2	10.1	4.7	0.5	26.1	9.5	4.9	0.4	18.9	10.8	3.3	0.7
	2 min	14.2	10.8	4.5	0.2	10.0	11.1	4.4	1.0	14.0	10.4	3.5	0.7
	1 min	15.4	9.3	3.4	0.9	11.4	7.4	2.7	0.3	13.3	8.3	1.4	1.2
63 ul	16 min	17.1	10.3	2.3	2.6	14.3	8.1	1.1	2.0	24.4	9.9	1.8	2.6
	8 min	16.6	9.5	1.2	0.7	12.4	7.2	1.4	0.3	19.4	8.8	0.0	0.2
	4 min	11.9	6.5	3.2	1.6	12.4	5.1	2.7	1.3	10.9	4.3	2.8	2.5
	2 min	12.3	5.1	0.1	1.8	9.2	3.7	0.4	1.5	10.1	2.6	0.5	1.7
	1 min	11.9	5.3	1.0	1.5	13.0	4.3	0.6	1.3	6.3	2.7	0.2	1.8
31 ul	16 min	10.9	6.8	1.2	1.9	7.9	4.7	0.6	1.4	6.0	4.2	1.3	1.5
	8 min	10.0	3.6	0.2	2.0	8.0	3.4	0.1	1.5	4.2	2.4	0.6	1.9
	4 min	6.4	3.7	1.7	1.2	5.6	2.9	1.0	0.7	2.8	1.7	1.3	0.6
	2 min	4.3	6.9	0.8	2.1	3.6	6.9	1.4	1.6	1.5	5.1	0.4	1.5
	1 min	3.8	1.2	0.8	0.3	4.2	0.5	0.7	0.2	0.1	0.3	0.6	0.1

Table 5 Signal to Noise Ratio for the HRRT Scanner

Siemens HRRT													
		1 vox				2 vox				4 vox			
		15:1	7.5:1	3.75:1	1.88:1	15:1	7.5:1	3.75:1	1.88:1	15:1	7.5:1	3.75:1	1.88:1
2000 ul	16 min	23.9	20.3	17.3	7.9	21.3	18.6	13.2	7.0	21.8	15.6	12.2	6.6
	8 min	19.9	18.6	14.6	8.0	20.3	14.5	14.2	7.2	18.6	17.0	8.1	5.7
	4 min	20.5	19.8	12.4	5.1	11.5	17.2	13.6	5.1	19.4	15.3	9.5	9.0
	2 min	18.4	15.7	12.2	7.3	13.6	20.5	11.7	6.2	12.8	12.5	8.7	4.6
	1 min	18.1	14.6	10.1	7.8	11.5	37.3	9.2	6.8	12.0	15.4	6.2	3.0
1000 ul	16 min	22.1	22.4	13.5	4.7	16.9	18.2	9.9	3.6	13.5	12.7	8.3	3.3
	8 min	17.4	18.9	11.4	5.0	16.3	14.9	10.9	4.7	15.5	9.7	8.3	3.0
	4 min	14.5	15.7	11.5	6.5	78.2	12.6	10.9	3.9	16.9	32.3	7.3	3.6
	2 min	17.8	16.2	11.0	5.1	13.7	13.7	9.8	4.4	11.3	12.8	7.9	2.0
	1 min	22.7	14.7	11.2	5.7	12.5	30.6	11.9	4.9	30.4	8.5	6.6	3.1
500 ul	16 min	21.7	18.0	11.3	3.3	14.2	10.5	9.3	3.4	49.3	32.5	11.0	3.3
	8 min	18.2	17.7	11.3	4.0	14.3	14.9	9.1	2.5	34.5	21.5	10.1	2.2
	4 min	16.4	16.9	8.4	2.5	18.7	12.5	7.3	1.9	32.1	14.3	7.2	0.2
	2 min	15.3	15.3	7.5	7.8	31.5	12.8	6.8	6.5	24.0	12.7	3.3	2.0
	1 min	16.3	13.9	6.7	3.4	18.0	9.4	7.7	3.8	19.7	11.1	1.5	1.4
250 ul	16 min	16.8	15.9	10.3	1.3	13.8	13.8	7.7	2.0	35.0	20.1	7.4	0.9
	8 min	16.2	15.1	7.9	1.8	9.2	15.2	7.0	1.6	23.4	15.3	3.0	0.5
	4 min	19.6	14.9	9.5	3.7	10.2	31.1	7.1	0.3	16.4	8.5	4.2	0.5
	2 min	17.8	14.5	7.2	4.8	15.3	13.4	6.5	3.0	10.6	9.4	4.9	0.8
	1 min	15.3	14.9	6.7	1.3	37.4	12.1	4.6	1.7	13.8	3.9	0.1	0.8
125 ul	16 min	17.8	12.6	4.7	0.6	12.0	10.2	3.0	0.4	24.7	11.1	2.7	1.6
	8 min	18.2	10.1	1.9	0.1	23.3	10.0	2.7	0.1	16.6	9.2	1.4	0.3
	4 min	12.7	11.3	4.6	0.1	15.3	20.5	4.8	0.8	11.5	6.8	3.0	1.3
	2 min	12.5	11.0	6.1	1.4	11.6	8.5	8.2	2.3	8.4	5.6	1.9	0.4
	1 min	11.3	7.7	3.9	2.5	14.1	7.3	2.3	0.8	3.6	1.6	0.6	1.3
63 ul	16 min	14.7	10.9	4.0	0.4	26.1	7.7	1.8	0.1	15.6	3.9	0.8	0.0
	8 min	15.8	7.1	2.9	0.5	17.4	5.3	2.1	1.0	12.0	2.6	0.2	0.7
	4 min	11.4	5.5	2.5	1.3	8.7	4.1	1.5	0.3	7.8	3.6	0.3	0.1
	2 min	7.6	3.3	3.0	1.1	4.9	2.4	1.9	0.7	2.6	0.5	0.3	0.5
	1 min	12.7	3.9	4.1	1.4	11.9	1.4	0.7	0.3	1.7	0.5	0.9	2.1
31 ul	16 min	6.1	4.4	3.6	0.5	4.0	1.9	3.8	1.1	3.1	0.0	2.5	0.2
	8 min	5.7	3.6	3.1	1.3	2.5	1.6	3.9	0.6	2.8	1.3	2.1	0.6
	4 min	4.0	2.7	0.1	0.4	0.6	1.5	0.2	0.5	1.2	0.8	0.4	1.0
	2 min	3.9	2.1	2.8	0.5	0.6	0.2	0.3	0.6	0.4	0.8	1.1	0.8
	1 min	4.1	9.7	2.1	1.2	0.1	3.2	0.9	0.2	0.4	2.2	2.0	0.9

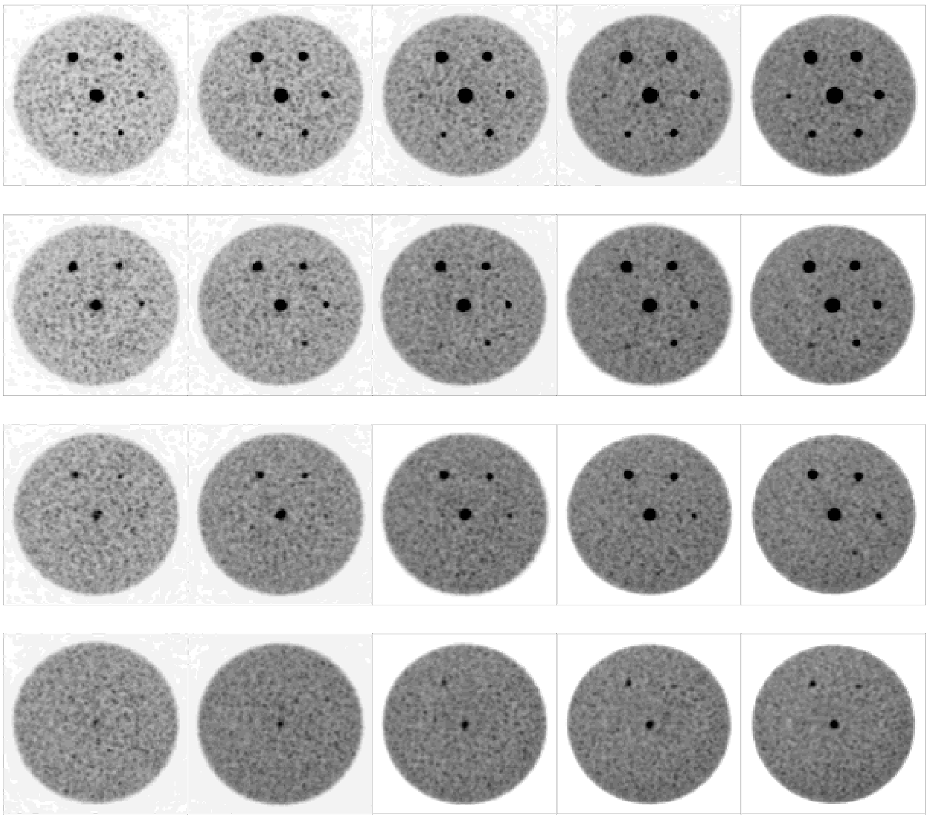

Fig. 2 Gemini TF, 2mm x 2mm x 2mm voxels

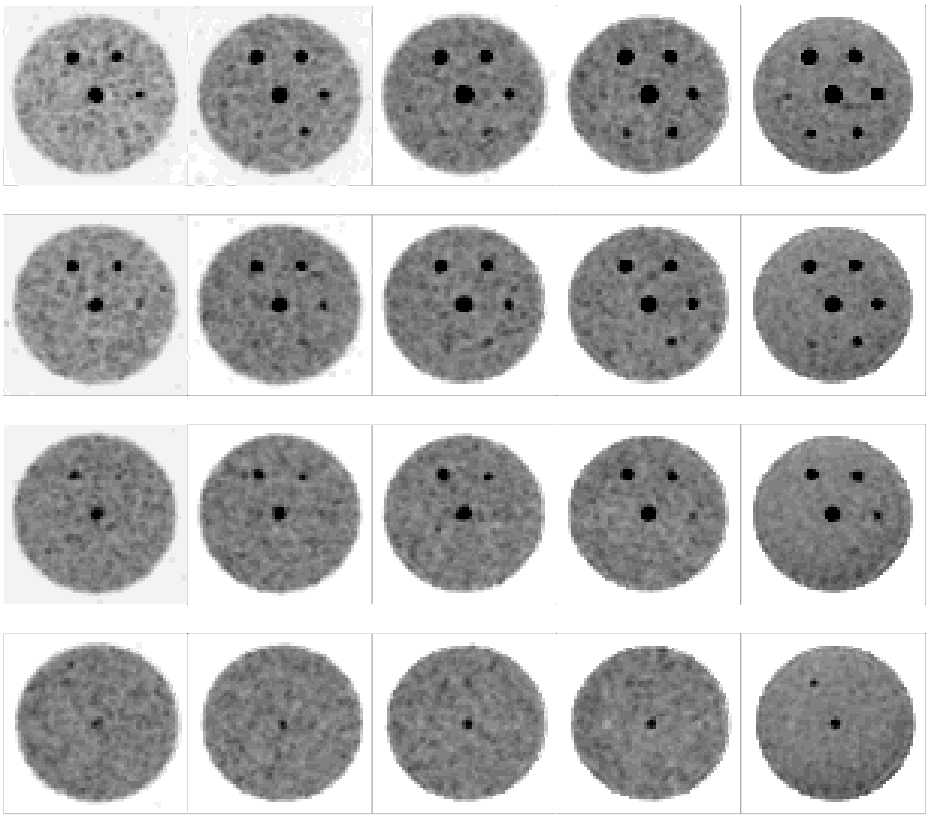

Fig. 3 Gemini TF, 4mm x 4mm x 4mm voxels

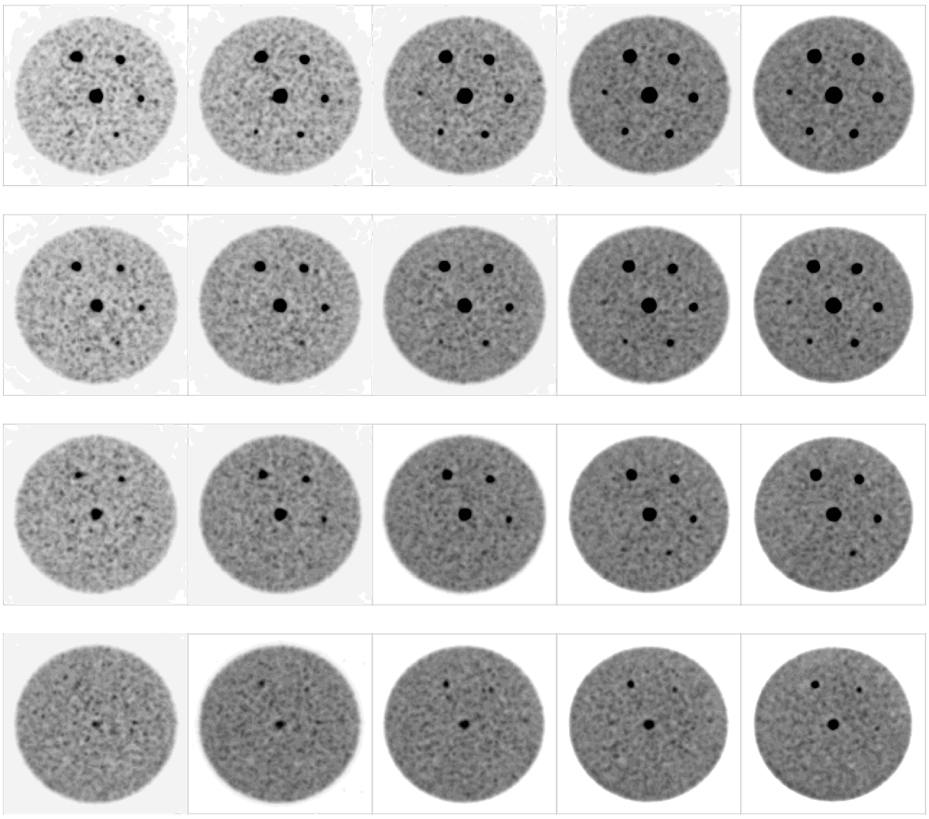

Fig. 4 Vereos, 1mm x 1mm x 1mm voxels

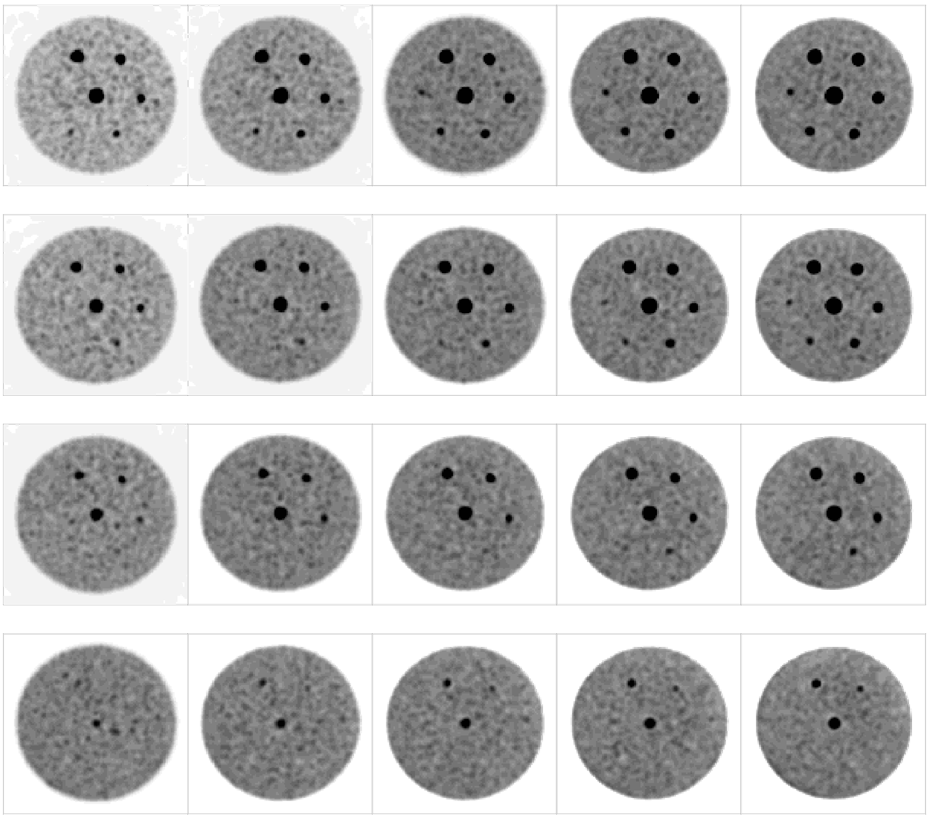

Fig. 5 Vereos, 2mm x 2mm x 2mm voxels

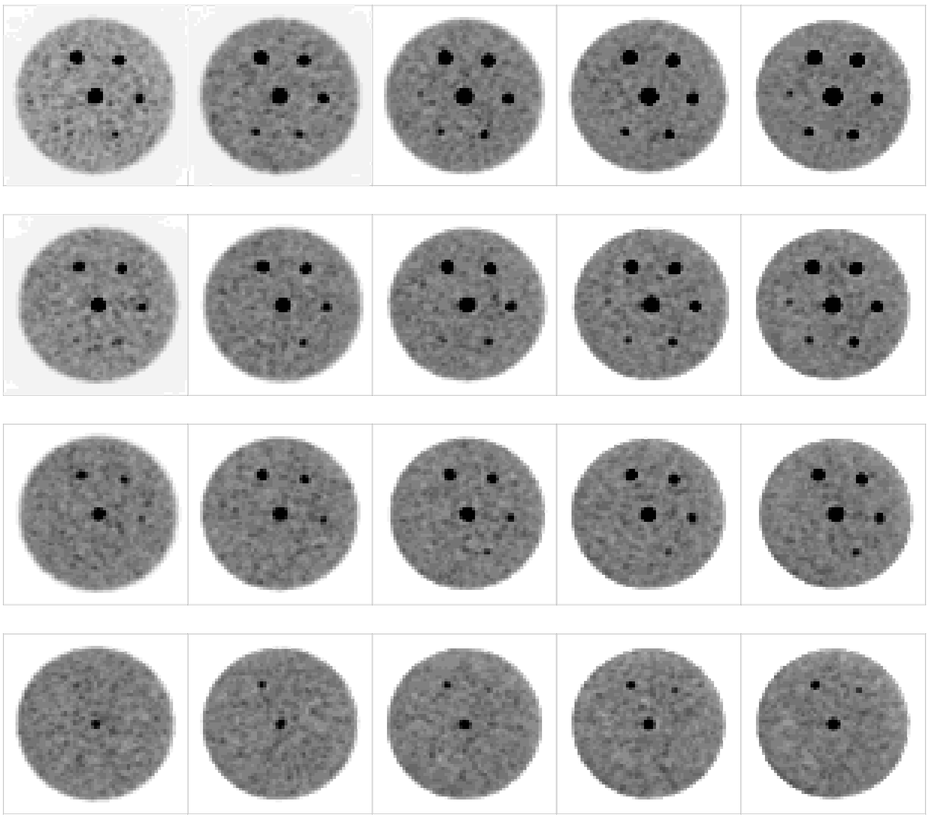

Fig. 6 Vereos, 4mm x 4mm x 4mm voxels

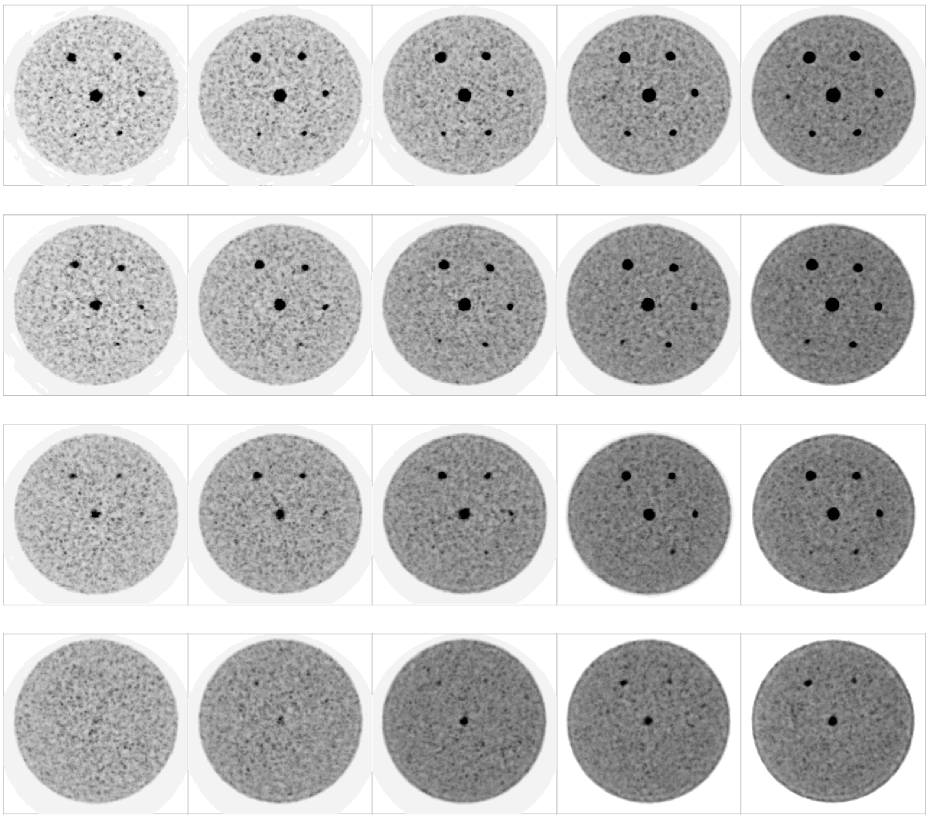

Fig. 7 Discovery 710, 1mm x 1mm x 3.27mm voxels

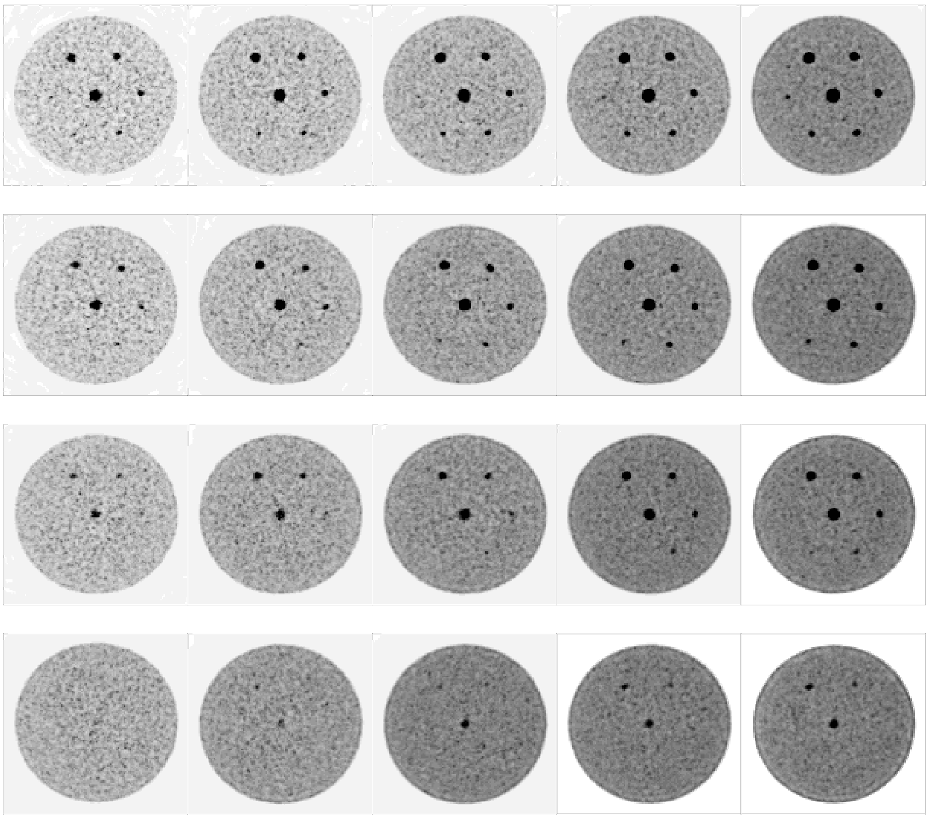

Fig. 8 Discovery 710, 2mm x 2mm x 3.27mm voxels

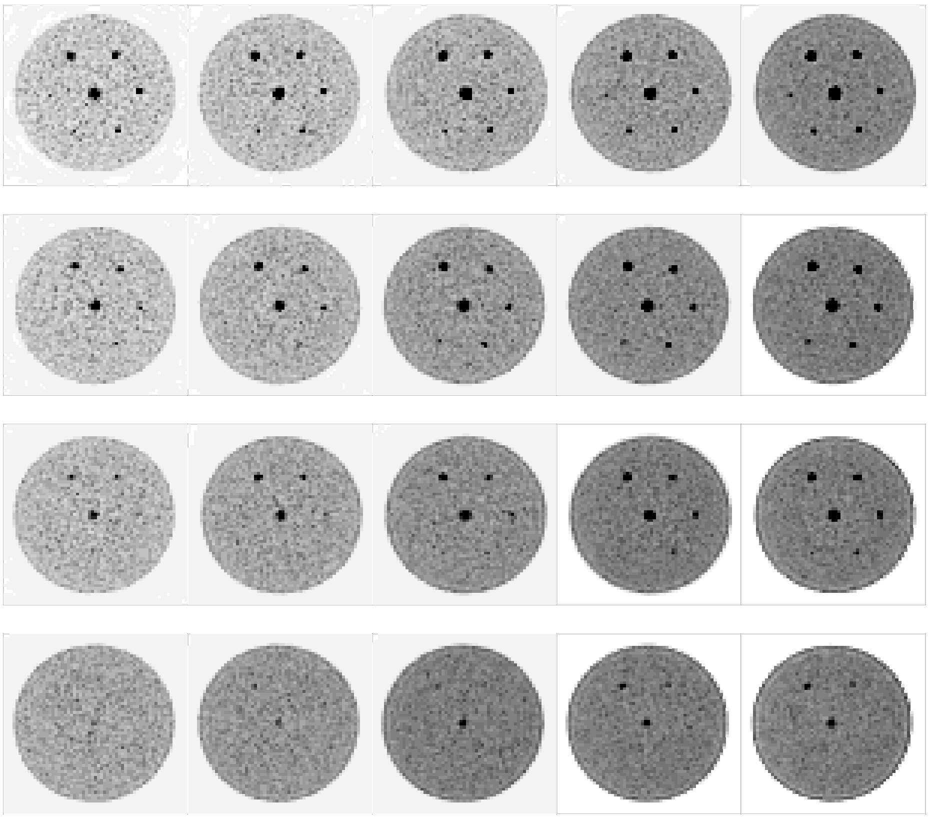

Fig. 9 Discovery 710, 4mm x 4mm x 3.27mm voxels

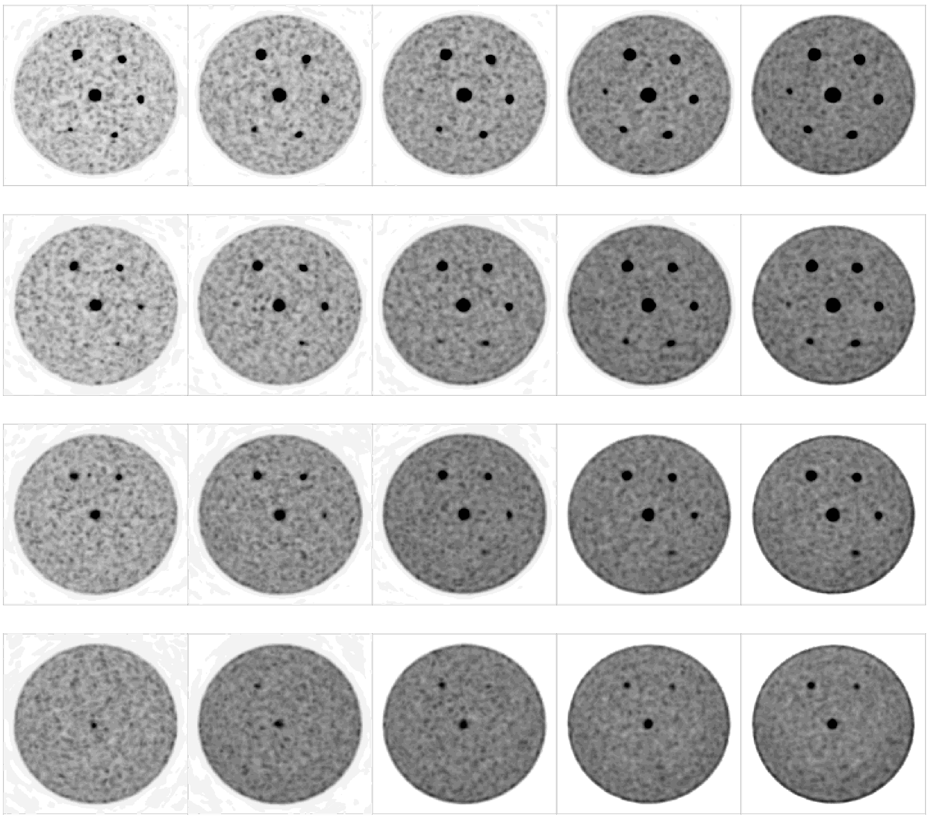

Fig. 10 Biograph mCT, 1.07mm x 1.07mm x 1mm voxels

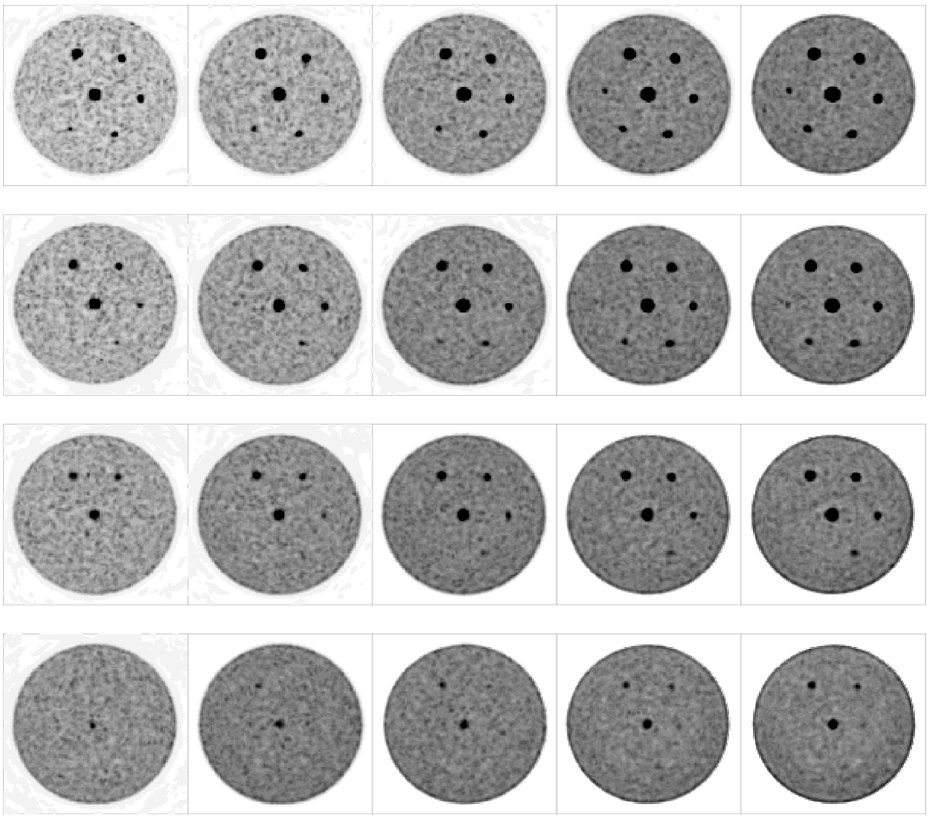

Fig. 11 Biograph mCT, 1.99mm x 1.99mm x 2mm voxels

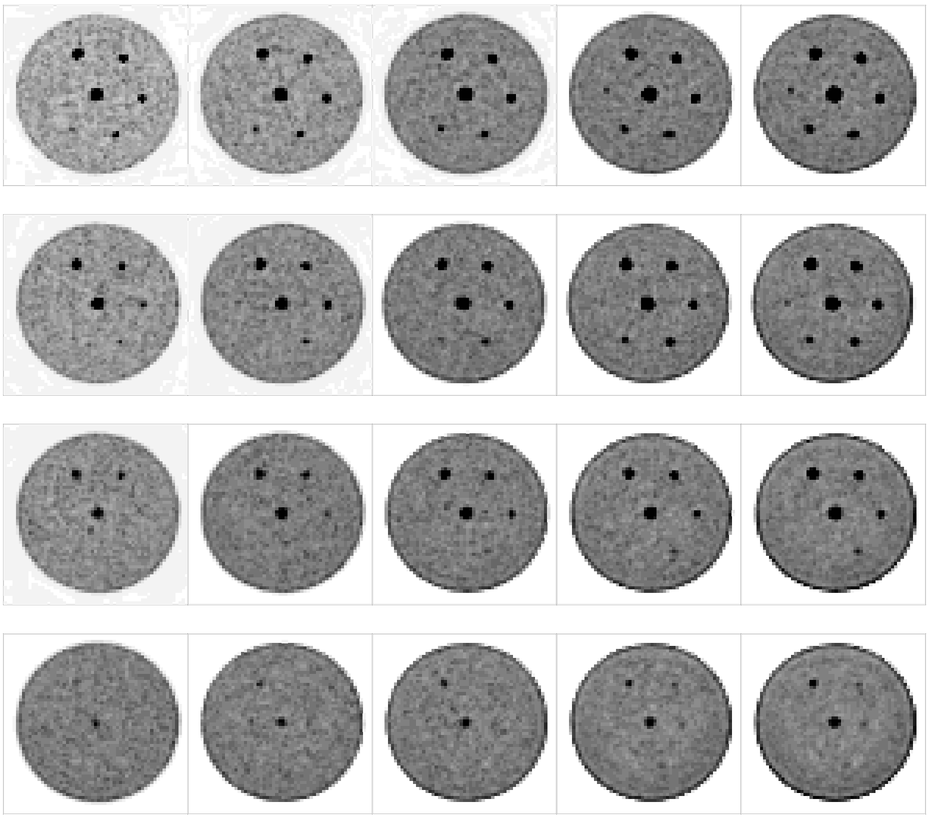

Fig. 12 Biograph mCT, 4.07mm x 4.07mm x 4mm voxels

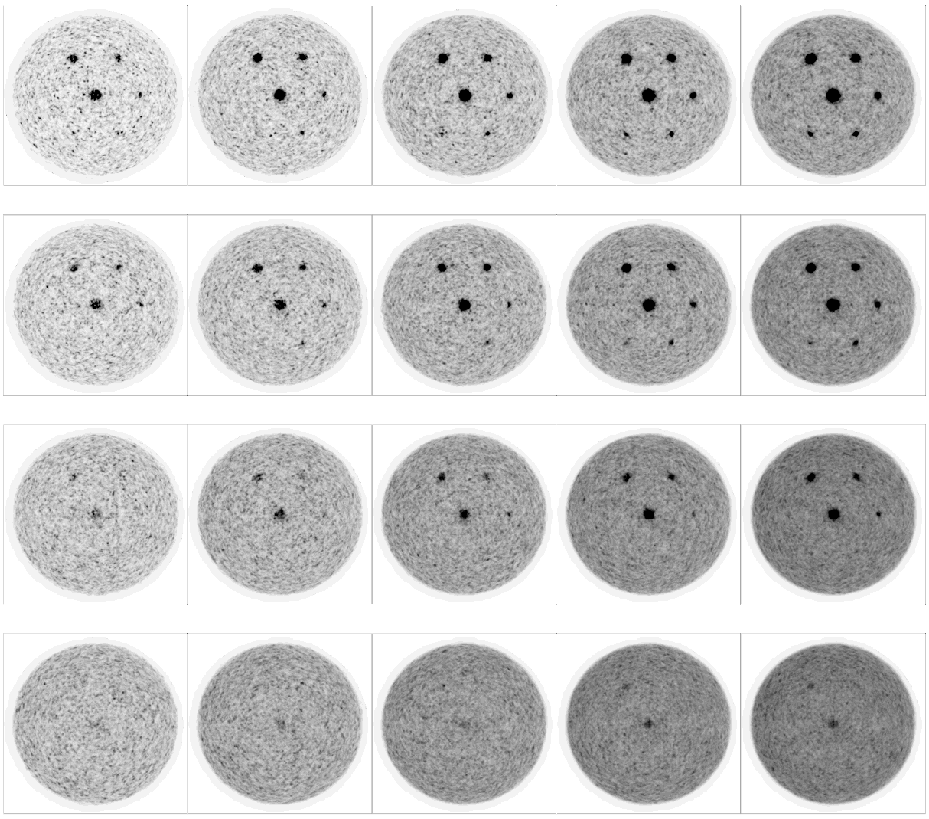

Fig. 13 HRRT, 1mm x 1mm x 1mm voxels

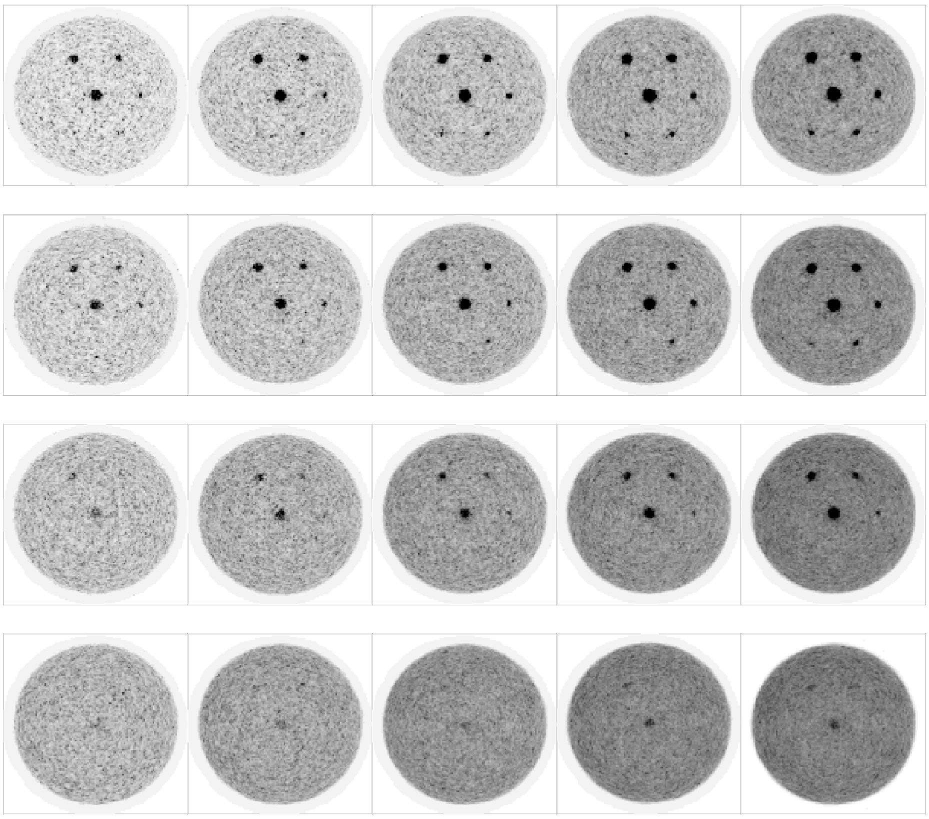

Fig. 14 HRRT, 2mm x 2mm x 2mm voxels

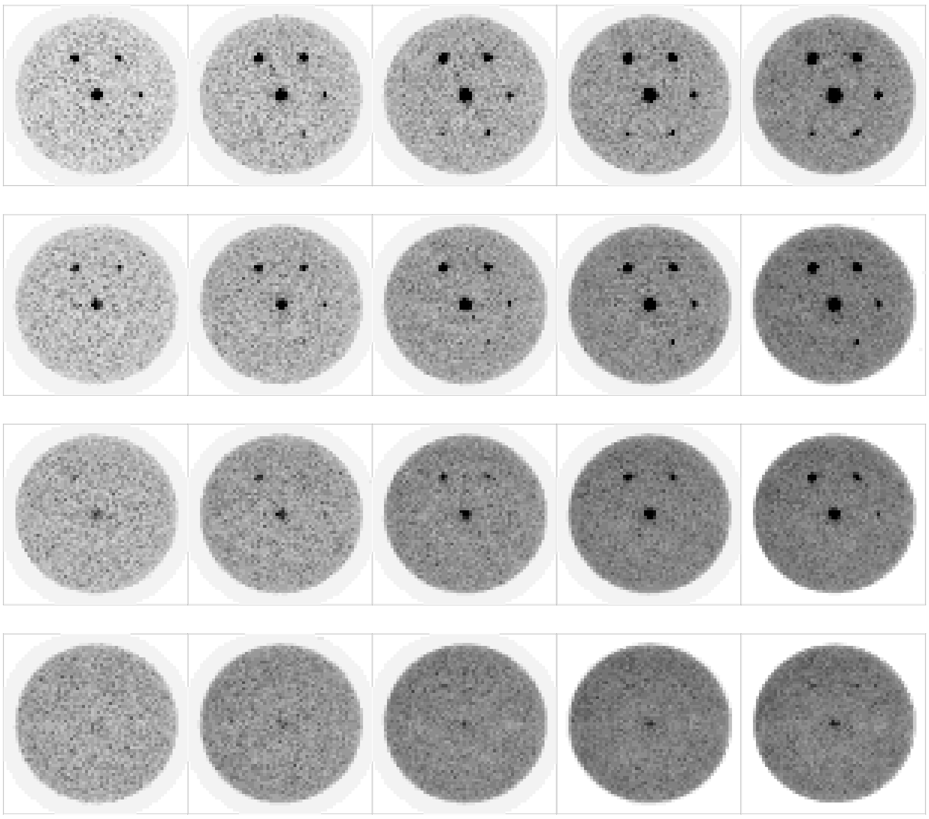

Fig. 15 HRRT, 4mm x 4mm x 4mm voxels
